# Characteristics and Outcomes of Children with Clinical History of Atopic *Versus* Non-atopic Asthma Admitted to a Tertiary Pediatric Intensive Care Unit

**DOI:** 10.2174/1874306401812010021

**Published:** 2018-05-31

**Authors:** Jamie Causey, Traci Gonzales, Aravind Yadav, Syed Hashmi, Wilfredo De Jesus-Rojas, Cindy Jon, Ikram Haque, Richard Johnston, James Stark, Katrina McBeth, Giuseppe Colasurdo, Ricardo Mosquera

**Affiliations:** 1 Department of Pediatrics Division of Pediatric Critical Care Medicine, University of Texas Health Science Center at Houston, Houston, USA; 2 Department of Pediatrics Division of Pulmonary Medicine, University of Texas Health Science Center at Houston, Houston, USA; 3 Department of Pediatrics, University of Texas Health Science Center at Houston, Houston, USA

**Keywords:** Atopic, Non-Atopic, Asthma, PICU, LOS, IQR

## Abstract

**Background:**

Children admitted to the Pediatric Intensive Care Unit (PICU) with status asthmaticus have variable clinical courses, and predicting their outcomes is challenging. Identifying characteristics in these patients that may require more intense intervention is important for clinical decision-making.

**Objective:**

This study sought to determine the characteristics and outcomes, specifically length of stay and mortality, of atopic *versus* non-atopic asthmatics admitted to a PICU with status asthmaticus.

**Methods:**

A retrospective study was conducted at a children’s hospital from November 1, 2008 to October 31, 2013. A total of 90 children admitted to the PICU were included in the analysis. Patients were divided into two groups based on the presence of specific historical data indicative of a clinical history of atopy. Children were considered to be atopic if they had a parental history of asthma, a personal history of eczema, or a combined history of wheezing (apart from colds) and allergic rhinitis (diagnosed by a medical provider). The median hospital Length Of Stay (LOS), PICU LOS, cardiopulmonary arrest, and mortality were compared between atopic and non-atopic asthma groups. Regression models were used to estimate the LOS stratified by atopic or non-atopic and by history of intubation in present hospitalization.

**Results:**

Median hospital LOS for atopic children was 5.9 days (IQR of 3.8-8.7) and 3.5 days (IQR of 2.2-5.5) for non-atopic asthmatics (z = 2.9, *p* = 0.0042). The median PICU LOS was 2.5 days (IQR 1.4-6.1) for atopic asthmatics and 1.6 days (IQR 1.1-2.4) for non-atopic asthmatics (z = 2.5, *p* = 0.0141). The median LOS was significantly higher for atopic intubated patients compared to non-atopic intubated patients (p=0.021). Although there was an increased tendency towards intubation in the atopic group, the difference was not significant. There was no significant difference in cardiopulmonary arrest or mortality.

**Conclusion:**

A clinical history of atopic asthma in children admitted to the PICU with status asthmaticus was associated with longer length of stays The longest LOS was observed when atopic patients required intubation.

## 1 INTRODUCTION

Asthma is one of the most common chronic diseases of childhood, affecting more than 7 million children in the United States [1]. Annually, there are at least 1.8 million emergency department visits, 439,000 hospital admissions with asthma as the first-listed diagnosis, and 3,630 annual childhood deaths attributed to asthma [2]. Approximately, 2-20% of all patients are admitted for acute asthma management to the intensive care unit with severe or life-threatening asthma [3] and up to one third of these patients require intubation during their acute exacerbation [4].

Children admitted to the pediatric intensive care unit (PICU) with near-fatal asthma exacerbations have variable clinical courses [5]. Predicting their outcomes, such as length of PICU or hospital stay, necessity of ventilator support, and mortality risk, is complicated. Early identification of children with specific characteristics or phenotypes that have a higher risk of requiring more intense intervention and portend a poorer outcome is important. Identification might lead to implementation of more aggressive treatment and different respiratory support strategies in the early stages of admission, and therefore lead to better patient outcomes

Epidemiological studies have suggested that there are several different asthma phenotypes and that there are clinically significant differences between atopic and non-atopic asthma phenotypes. The phenotypes have differences in their underlying disease process, which can affect outcomes and require different treatment strategies [6, 7]. Results are inconclusive regarding which phenotype has more severe prognosis. Unfortunately, the outcomes and treatment responses for different phenotypes in children with life-threatening asthma exacerbation require PICU admission have not yet been described.

Atopic asthma is the more predominant phenotype found in children. Characteristics of atopic asthma include a family history of atopy, high serum IgE, and positive reactivity on allergy testing. Treatment with corticosteroids is usually beneficial [8]. Non-atopic asthma is associated with a negative family or personal history of atopy or allergic response and neutrophilic airway inflammation that appears to be relatively resistant to corticosteroid therapy [8]. It could carry a worse prognosis due to the potential resistance to the treatment, especially when admitted to the hospital with an asthma exacerbation [8, 9]. However, other studies have demonstrated that asthmatic children with the atopic phenotype have a more severe illness course when compared to their non-atopic counterparts [10].

While the characteristics of atopic asthma are well defined in the literature, diagnostic components are not always readily available in the clinical setting. It is not generally feasible to perform allergen reactivity testing in the acute care setting. Likewise, serum IgE and eosinophil count, if obtained, could be altered due to acute illness or medications given during exacerbation. Based on the high predictive value of the Asthma Predictive Index (API) [11], a tool that is frequently used in the clinical setting to determine risk of atopic asthma, we chose similar characteristics to define atopic asthma in this study.

Using a retrospective chart review in an urban, tertiary-care PICU, this study compared the characteristics and clinical outcomes of patients with atopic phenotype to those with non-atopic phenotype. We hypothesized that patients with non-atopic asthma would have an increased PICU and hospital length of stay when compared to patients with atopic asthma.

## 2 METHODS

### 2.1 Study Population

This study utilized a historical or retrospective cohort design whereby a retrospective chart review was conducted at Children’s Memorial Hermann Hospital, an urban, university-affiliated, tertiary pediatric intensive care unit (PICU) in Houston, Texas. A cohort of children (aged 5 -18 years), that had been admitted to the PICU for an acute asthma exacerbation from 1 November 2008 through 31 October 2013, were identified by the following ICD-9^TM^ codes: 493.01 Extrinsic asthma unspecified, 493.02 Extrinsic asthma with (acute) exacerbation, 493.10 Intrinsic asthma unspecified, 493.11 Intrinsic asthma with (acute) exacerbation, 492.20 Chronic obstructive asthma unspecified, 493.21 Chronic obstructive asthma with status asthmaticus, 493.22 Chronic obstructive asthma with acute exacerbation, 493.82 Cough variant asthma, 493.90 Asthma unspecified, 493.91 Asthma unspecified with status asthmaticus, 493.92 Asthma unspecified with (acute) exacerbation, and 786.07 Wheezing.

Patients were divided into two groups based on the presence of specific historical data indicative of a clinical history of atopy. Children were considered to be atopic if they had a parental history of asthma, a personal history of eczema, or a combined history of wheezing (apart from colds) and allergic rhinitis (diagnosed by a medical provider). All other children were categorized as being non-atopic. Patients were excluded if they had a history of tracheomalacia, prematurity (birth at < 32 weeks gestational age), bronchopulmonary dysplasia, or if their primary reason for hospitalization was due to a cause other than an asthma exacerbation.

### 2.2 Recorded Data

Patient demographics abstracted from medical records included age, gender, and race. Additional data included body-mass index, family history of asthma and atopic symptoms, co-morbid condition(s), history of prior ICU admissions, white cell count with differential, and if a personal history of allergic rhinitis, wheezing, or eczema was present. The electronic medical records were reviewed for hospital and ICU length of stay, discharge disposition, the occurrence of cardiopulmonary arrest prior to the hospitalization, and the occurrence and duration of intubation requiring mechanical ventilation.

### 2.3 Statistical Analysis

Data were collected and described based on the data type and distribution. The medians (with interquartile range, IQR) were reported for non-normally distributed continuous data. The means (with 95% Confidence Intervals, 95% CI) were reported for normally distributed continuous data, and the frequencies (with percentages) were reported for categorical data. The Mann-Whitney rank sum test was used to compare the distributions of continuous data, while the Fisher exact or Chi-square test was used to compare the categorical data. The median time to discharge from hospital was compared using a Wilcoxon rank sum test. The association of various factors with time to discharge (length of stay) was performed using Cox regression models (time-to-event analysis) with time to discharge from hospital as the outcome of interest. Multivariate models assessing length of stay included the presence of atopy as an independent variable as well as other effect modifiers, such as intubation, race/ethnicity, gender, history of cardiopulmonary arrest, and whether or not a pulmonologist was consulted. A separate set of these multivariate models were also run that included terms to assess interactive effects between atopy and intubation. The analyses were also performed after excluding patients that had a history of a cardiopulmonary arrest. The Hazard Ratios (HR) from these regression models reflect an increased likelihood of the event (discharge from hospital or PICU) occurring for HR higher than 1.0 and a decreased likelihood of the event (discharge) occurring for HR lower than 1.0.

*A priori* power calculations suggested that a sample size of 150 subjects (1:1 ratio) would be sufficient to detect, at a Type I error rate of 5% and power of 80%, a difference in hospital length of stay between atopic and non-atopic patients if the median LOS was 6 days in the non-atopic patients and was less than 4.3 days or more than 8.3 days in the atopic patients. A two-tailed *p-*value of 0.05 was considered statistically significant during analysis. All analyses were performed using STATA (v. 14, StataCorp, College Station, TX).

## 3 RESULTS

**Fig. (1):**
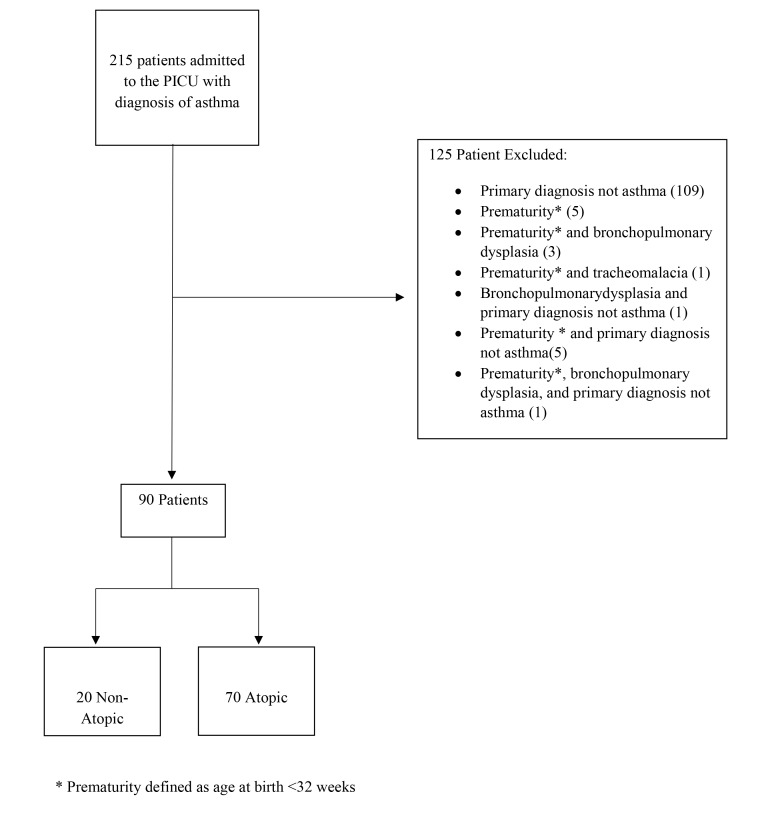
Prematurity defined as age at birth < 32 weeks.

There were 215 patients between the ages of 5 and 18, with a diagnosis of asthma, who were admitted to the PICU during their hospitalization. From this, 125 patients met the exclusion criteria (Fig. **1**). Of the 90 remaining children, 20 (22%) were found to be non-atopic, while 70 patients (78%) were atopic (Table **1**). On univariate analysis, the two groups had similar baseline distributions with respect to age, gender, and body mass index. There were some differences in ethnic distribution between the two groups, where in comparison to non-atopic patients, the atopic patients were more commonly identified as Black (69% *vs* 40%) and less commonly as Hispanic (17% *vs* 35%), though the overall differences in ethnic distribution were not statistically significant (p=0.061). All factors (family history of asthma, personal history of eczema and a history of allergic rhinitis) that determined classification of the patients as atopic or non-atopic were independently different between the two groups (*p*<0.02).

The length of stay was significantly shorter in the non-atopic patients than in the atopic patients (Table **2**). Median hospital length-of-stay was 3.5 days (IQR 2.2-5.5) in the non-atopic group, and 5.9 days (IQR 3.9-8.7) in the atopic group (p=0.004). The same trend was observed when comparing PICU length-of-stay between the two groups, with the non-atopic patients having a shorter median PICU length-of-stay of 1.6 days (IQR 1.1-2.4) compared to the atopic group with a median PICU stay of 2.5 days (IQR 1.4-6.1) (*p* = 0.010). When comparing intubation rates between the groups, 44% of atopic patients required intubation, while 25% of non atopics required the same. Although there was an increased tendency towards intubation in the atopic group, the difference was not found to be statistically significant (Table **2**).

There were multiple patients in both groups who presented with out-of-hospital cardiopulmonary arrest secondary to their acute asthma exacerbation. In the atopic group, eight patients (11%) were presented to the hospital in cardiopulmonary arrest. Three of those eight patients (37.5%) had severe neurologic injury and subsequently met criteria for death by neurologic determination, so life support was withdrawn. Five of the eight patients (62.5%) sustained significant neurologic injury which prolonged their hospitalizations, ICU time, and ventilator time. In the non-atopic group, two patients (10%) were presented in cardiopulmonary arrest, one of whom was declared brain dead.

Since these patients with cardiopulmonary arrest had hospitalization durations that reflected factors other than the severity of their lung disease, a subsequent analysis was performed excluding those patients. The overall length of stay in hospital was still shorter for the non-atopic group (median: 3.5 days, IQR: 2.0-5.0) than the atopic group (median: 5.6 days. IQR: 3.8-7.4) (p=0.011). This trend was again replicated with regards to PICU length of stay with non-atopic patients (median: 1.5 days, IQR: 1.0-2.3) spending fewer days in the PICU than atopic patients (median: 2.2 days, IQR: 1.3-3.9) (p=0.023).

The independent effect of atopy on length of stay was evident also in the multivariate models and suggested that atopic patients had a lower likelihood of discharge from hospital (HR: 0.21; 95% CI: 0.07 – 0.64) and PICU (HR: 0.23; 95% CI: 0.08 – 0.70) compared to the non-atopic patients. Additionally, intubated patients compared to non-intubated patients (HR_hospital: 0.28, 95% CI: 0.08 – 0.90; HR_PICU: 0.21, 95% CI: 0.06 – 0.72) and Black patients compared to non-Hispanic whites (HR_hospital: 0.17, 95% CI: 0.05 – 0.58; HR_PICU: 0.25, 95% CI: 0.08 – 0.81) were also less likely to be discharged. These hazard ratios were adjusted for all of the other effect modifiers mentioned in the Methods section.

**Fig. (2):**
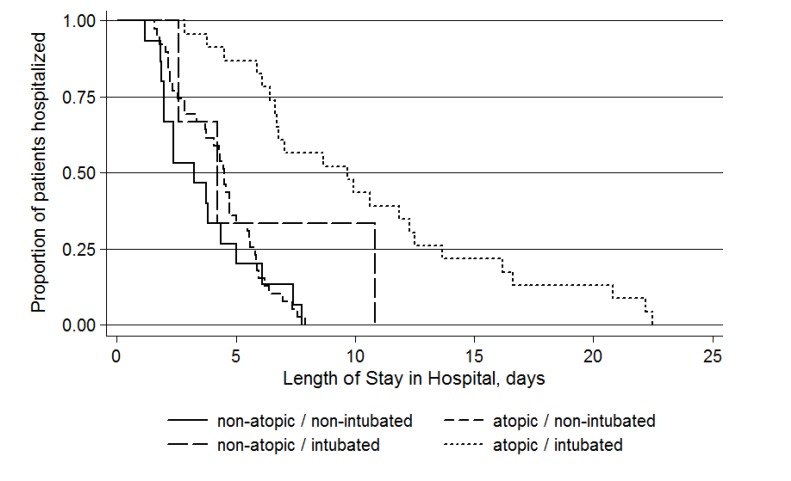
Proportion of patients hospitalized.

To assess for effect modification and/or statistical interaction, regression models stratified by the presence or absence of atopy and/or intubation and regression models including interaction terms were assessed, respectively. No evidence of statistical interaction was observed from the latter models. However, the stratified analysis showed a differential effect on length of stay depending on whether or not a history of atopy was present and/or intubation occurred (Table **3** and Fig. **2**).

The median time was 3.3 days (IQR: 2.0 – 5.0) for hospital stays among non-atopic patients that did not get intubated. This was not statistically different for the median hospital stay for non-atopic patients who got intubated (4.2 days; IQR: 2.6 – 6.0; p=0.238) or atopic patients who did not get intubated (4.5 days; IQR: 2.5 – 5.8; p=0.179). However, atopic patients who got intubated had significantly longer hospital stays (9.9 days; IWR: 6.4 – 16.6) compared to all the other groups (Table **3**).

A similar pattern was observed with time spent in the PICU, with the atopic intubated patients having the longest stays. There was no difference between the median time in the PICU among the non-intubated patients regardless of whether they were non-atopic (1.5 days; IQR: 0.9-1.9) or atopic (1.7 days; IQR: 1.2-2.4) (p=0.275). However, the non-atopic non-intubated patients had significantly shorter PICU stays compared to the intubated patients, both non-atopic (2.6 days; IQR: 2.4 – 4.9; p=0.016) and atopic (7.9 days; IQR: 3.5 – 11.2; p<0.001). When atopic and non-atopic patients who were intubated were compared to each other, the median length of stay in the PICU was longer for the atopic patients (7.9 days *vs* 2.6 days, respectively) but it failed to achieve statistical significance (p=0.052).

Similar trends were observed for the time-to-event analyses for both hospital and PICU stays. At any time during their stay, the patients who were atopic and intubated had a significantly lower likelihood of discharge from the hospital (HR: 0.14, 95% CI: 0.05-0.35) or the PICU (HR: 0.06, 95% CI: 0.02 – 0.18) compared to the non-atopic non-intubated patients. Additionally, when the analysis was limited to only the intubated patients, the atopic patients again had a significantly lower likelihood of discharge from the hospital (HR: 0.25, 95% CI: 0.09-0.69) and the PICU (HR: 0.29, 95% CI: 0.10 – 0.81) compared to the non-atopic patients.

All these analyses were repeated after exclusion of 10 patients with cardiopulmonary arrest to account for any effect that cardiopulmonary arrest had on length of stay. All these 10 patients had been intubated. Therefore, results did not change for the comparisons by atopy among the non-intubated patients. However, among the intubated patients, only 3 non-atopic and 23 atopic patients were left in the analysis. Although the median times in these 23 atopic patients were nearly double for both hospital stay (9.7 *vs* 4.2 days) and PICU stay (5.8 *vs* 2.4 days) when compared to the 3 non-atopic patients (Table **3**), they failed to reach statistical significance (*p*=0.138 and *p*=0.185, respectively).

## 4 DISCUSSION

The results of this study indicate that atopic asthmatic children required longer hospitalization when compared to those without atopy. On average, atopic asthmatic children required 2 days longer total hospital course and 1 day longer in the PICU, with significantly longer length of stay in atopic children who were intubated.

Our study had a significantly higher percentage of children in the atopic asthmatic group (n = 70, 78%) when compared to the non-atopic asthmatics (n = 20, 22%). Published data regarding proportion of asthma cases attributable to atopy in children has been noted to be 25-63%, with more recent studies, suggesting prevalence to be in the mid-30s [12, 13]. The discrepancy in our patient distribution may be attributable to the definition of atopic asthma that we applied, which is primarily based on clinical characteristics per parental recall as documented in the medical record. Most studies utilize objective findings to establish atopy, such as skin prick testing, which is not feasible in the acute care setting. Instead, we relied on simple questions that could quickly assess the risk for atopy. In most clinical practice settings, clinical history is a more practical, more cost-effective, and easier way to distinguish clinical asthma phenotypes.

Our findings must be interpreted in the context of the following limitations. This is a retrospective and cohort study design; data collection was limited to the information available in the medical records. While the primary outcomes regarding length of stay had objective findings, historical elements obtained from chart review were limited to available medical record documentation. As such, atopic features not mentioned in the patient’s chart could not exclude the possibility of atopy. In addition, the history of atopy was dependent on the patient and family affirmation and not necessarily based on physician diagnosed parameters. Clinical history regarding baseline asthma severity, asthma control, adherence with controller asthma therapy, pattern of beta agonist use, and potential signs of remodeling due to airway inflammation were not consistently available. The severity of the patient’s asthma and adherence pattern influences the level of lung inflammation and could have affected the outcomes within the study groups. In addition, our sample size was small, it could be that a large sample size could have resulted in a significant difference between the atopic and non-atopic groups.

Future prospective research is needed in order to confirm these results.

## CONCLUSION

Children with a clinical history of atopic asthma had longer duration of PICU care and hospitalization compared to patients with a negative clinical history of atopic asthma. For intubated children, the length of stay was longer if the patient had atopic asthma. Hence, intubation may be associated with higher risks in this population.

## Figures and Tables

**Table 1:** Demographic and medical history comparison between patients with non-atopic and atopic asthma.

	**Non-atopic (n=20)**	**Atopic (n=70)**	**p-value**
**Age at admission, years, mean (sd)**	**9.7%(3.6)**	**9.8% (2.9)**	**0.712**
**Gender male, % (n)**	**60% (12)**	**60%(42)**	**1**
**Ethnicity % (n)**			
**Black**	**40% (8)**	**69% (48)**	**0.061**
**Non-Hispanic white**	**15% (3)**	**11% (8)**	
**Hispanic**	**35% (7)**	**17% (12)**	
**Other**	**10% (2)**	**3% (2)**	
**Body mass index, median (IQR)**	**18.8 (15.6 - 22.2)**	**18.9 (16.1 - 22.8)**	**0.921**
**Cardiac arrest prior to arrival at hospital, % (n)**	**10% (2)**	**11% (8)**	**1**
**Family history of asthma, % (n) ^a^**	**0% (0)**	**80% (50)**	**<0.001**
**History of eczema, % (n) ^a^**	**0% (0)**	**30% (21)**	**0.003**
**History of allergic rhinitis, % (n) ^a^**	**35% (7)**	**66% (46)**	**0.02**
**History of wheezing or nighttime coughing, % (n)**	**25% (5)**	**42% (28)**	**0.2**
**Previous asthma-related events:**			
**PICU admission, % (n)**	**15% (3)**	**26% (18)**	**0.385**
**Intubation, % (n)**	**5% (1)**	**19% (13)**	**0.179**
**Days of symptoms prior to admission, median (IQR)**	**2.5 (1 - 4.5)**	**2 (1 - 3)**	**0.16**

Family history of asthma, personal history of eczema and a history of allergic rhinitis were factors that determined classification of the patients as atopic or non-atopic.

**Table 2:** Comparison of clinical parameters during current hospital admission.

-	**Non-Atopic (n=20)**	**Atopic (n=70)**	**p-Value**
**Intubated, % (n)**	**25% (5)**	**44% (31)**	**0.195**
**Intubation prior to local admission, % (n) of intubated patients**	**80% (4)**	**87% (27)**	**0.549**
**Duration of mechanical ventilation, hours, median (IQR)**	**62 (49 - 86)**	**130 (49 - 237)**	**0.293**
**Azithromycin administered % (n)**	**85% (17)**	**91% (64)**	**0.41**
**Initial WBC, x1000 cells, median (IQR)**	**12.6 (9.9 - 16.3)**	**13.6 (10.7 - 17.8)**	**0.536**
**Length of stay, days, median (IQR)**			
**Hospital**	**3.5 (2.2 - 5.5)**	**5.9 (2.8 - 8.7)**	**0.004**
**PICU**	**1.6 (1.1 - 2.4)**	**2.5 (1.4 - 6.1)**	**0.016**
**Died in hospital, % (n)**	**5% (1)**	**4% (3)**	**0.641**

**Table 3:** Length of hospital and PICU stay stratified by presence or absence of atopy and intubation.

Patient Atopic?	No	No	Yes	Yes
Patient intubated?	No	Yes	No	Yes
**All patients (n=90)**				
sample size	15	5	39	31
Length of hospital stay, days, median (IQR)	3.3 (2.0 - 5.0) **	4.2 (2.6 - 6.0) *	4.5 (2.5 - 5.8) **	9.9 (6.4 - 16.6)
Length of PICU stay, days, median (IQR)	1.5 (0.9 - 1.9) **	2.6 (2.4 - 4.9) †	1.7 (1.2 - 2.4) **	7.9 (3.4 - 11.2)
**Patient with no history of cardiopulmonary arrest (n=80)**				
sample size	15	3	39	23
Length of hospital stay, days, median (IQR)	3.3 (2.0 - 5.0) **	4.2 (2.6 - 10.8)	4.5 (2.5 - 5.8) **	9.7 (6.4 - 13.7)
Length of PICU stay, days, median (IQR)	1.5 (0.9 - 1.9) **	2.4 (1.3 - 8.1)	1.7 (1.2 - 2.4) **	5.8 (3.0 - 9.9)

** *p*<0.001 for Wilcoxon rank sum test when comparing to atopic-intubated patients * *p*<0.05 for Wilcoxon rank sum test when comparing to atopic-intubated patients † *p*=0.52 for Wilcoxon rank sum test when comparing to atopic-intubated patients

## LIST OF ABBREVIATIONS

PICU: = Pediatric intensive care unit
LOS: = Length of stay
IQR: = Inter quartile range
CI: = Confidence Interval
HR: = Hazard Ratio
